# Oligo-FISH Can Identify Chromosomes and Distinguish *Hippophaë rhamnoides* L. Taxa

**DOI:** 10.3390/genes13020195

**Published:** 2022-01-22

**Authors:** Xiaomei Luo, Juncheng Liu, Zhoujian He

**Affiliations:** College of Forestry, Sichuan Agricultural University, Huimin Road 211, Wenjiang District, Chengdu 611130, China; juhnson@foxmail.com (J.L.); hezhouj@163.com (Z.H.)

**Keywords:** *Hippophaë rhamnoides* L., oligo-FISH system, cytogenetic analysis, chromosomes, (TTG)_6_

## Abstract

Oligo-fluorescence in situ hybridization (FISH) facilitates precise chromosome identification and comparative cytogenetic analysis. Detection of autosomal chromosomes of *Hippophaë rhamnoides* has not been achieved using oligonucleotide sequences. Here, the chromosomes of five *H. rhamnoides* taxa in the mitotic metaphase and mitotic metaphase to anaphase were detected using the oligo-FISH probes (AG_3_T_3_)_3_, 5S rDNA, and (TTG)_6_. In total, 24 small chromosomes were clearly observed in the mitotic metaphase (0.89–3.03 μm), whereas 24–48 small chromosomes were observed in the mitotic metaphase to anaphase (0.94–3.10 μm). The signal number and intensity of (AG_3_T_3_)_3_, 5S rDNA, and (TTG)_6_ in the mitotic metaphase to anaphase chromosomes were nearly consistent with those in the mitotic metaphase chromosomes when the two split chromosomes were integrated as one unit. Of note, 14 chromosomes (there is a high chance that sex chromosomes are included) were exclusively identified by (AG_3_T_3_)_3_, 5S rDNA, and (TTG)_6_. The other 10 also showed a terminal signal with (AG_3_T_3_)_3_. Moreover, these oligo-probes were able to distinguish one wild *H. rhamnoides* taxon from four *H. rhamnoides* taxa. These chromosome identification and taxa differentiation data will help in elucidating visual and elaborate physical mapping and guide breeders’ utilization of wild resources of *H. rhamnoides*.

## 1. Introduction

*Hippophaë rhamnoides* L. (Elaeagnaceae), also known as sea buckthorn, is a spiny deciduous shrub or small tree [1]. This species originated and migrated from the Qinghai–Tibet Plateau and adjacent regions [2]. Its natural habitats include severe environments with excessive salinity, drought, cold, and heat [3]. *H. rhamnoides* is known for its nutritional, medicinal, and ecological values [4]; it has been shown to improve the health of consumers. Moreover, its berries, which are edible, are used as a general body-toning agent [3]. *H. rhamnoides*, and its processed products, are potentially nontoxic when consumed by humans as a food or as a dietary supplement [5]. Thus, the ecological and commercial values of *H. rhamnoides* have drawn the attention of researchers for centuries [6]. Furthermore, an increase in its demand has prompted the fine breeding of various cultivars with genetic improvements to achieve high productivity and quality.

The systematic treatment of *H. rhamnoides* has been controversial. Studies have reported inconsistent findings with respect to the number of *H. rhamnoides* subspecies, for example, two subspecies [7], three subspecies [8], six subspecies [9], eight subspecies [10], and nine subspecies [11]. The treatment of *Hippophaë rhamnoides* ssp. *sinensis* Rousi has been supported by the findings of Rousi [10] and Bartish et al. [12]. To date, the WFO [13] has shown that *H. rhamnoides* comprises three accepted subspecies, four unresolved subspecies, and one accepted variety. *H. rhamnoides* ssp. *sinensis* is an unresolved subspecies with one of the largest distribution ranges. Moreover, considering that abundant morphological variations have been described within the subspecies [12,14], it is critical to identify the genetic basis of these variations to facilitate the selection of superior cultivars from wild *H. rhamnoides* ssp. *sinensis*.

*Hippophaë rhamnoides* taxa are often misidentified owing to similarities in their vegetative morphology. Furthermore, the fruits of different species are labeled with the same name and are primarily sold or used in dried form or as powders. Therefore, different taxa cannot be identified based on only morphological characteristics, and accurate identification methods are needed to avoid misidentification and misuse. All *Hippophaë* species have been successfully identified by DNA barcoding, and four *H. rhamnoides* subspecies have also been differentiated using *ITS*2 and *psb*A-*trn*H [15]. The male/female plants of *H. rhamnoides* have been identified using inter-simple sequence repeat [16] and fluorescence in situ hybridization (FISH) [17]. However, none of the other molecular cytogenetic technologies can be used to identify *H. rhamnoides*, thus limiting investigations on its identification and characterization.

Oligos designed from conserved DNA sequences from one species, particularly from part/whole/multiple chromosomes, can be precisely identified from genetically related species, thereby allowing comparative cytogenetic mapping of these species. These oligonucleotide sequences can then be readily produced and tagged with fluorescent markers for use as oligo-probes in FISH [18]. Species identification based on such oligo-probes has been reported in an increasing number of plant species, such as *Avena* L. species [19], *Arachis hypogaea* L. [20], *Saccharum spontaneum* L. [21], *Citrus* L. species [22], *Citrus sinensis* (L.) Osbeck × *Poncirus trifoliata* (L.) Raf., CC [23], *Populus* L. species [24], *Strobus* Opiz species [25], and *Pinus* L. species [26]. However, information regarding *H. rhamnoides* is limited. Chromosome identification remains a major challenge in *H. rhamnoides* with small chromosomes. In the present study, we aimed to use three oligo-probes––(AG_3_T_3_)_3_, 5S rDNA, and (TTG)_6_—to identify *H. rhamnoides* chromosomes simultaneously in a single round of FISH.

## 2. Materials and Methods

The seeds of five *H. rhamnoides* taxa were used in this study; three *H. rhamnoides* cultivars (‘Shenqiuhong’, ‘Zhuangyuanhuang’, and ‘Wucifeng’) were collected from Hebei Province in China, one cultural *H. rhamnoides* ssp. *sinensis* was collected from Liaoning Province in China, and one wild *H. rhamnoides* ssp. *sinensis* was collected from Sichuan Province in China.

### 2.1. Oligo-Probe Preparation

The probe of the telomere (AG_3_T_3_)_3_ repeat sequence 5′-AGGGTTTAGGGTTTAGGGTTT-3′ originated from *Zea mays* L. [27] and was developed in *Berberis diaphana* Maxim. and *Berberis soulieana* Schneid. [28], *Fraxinus pennsylvanica* Marsh., *Syringa oblata* Ait., *Ligustrum lucidum* Lindl., *Ligustrum* × *vicaryi* Rehder [29], *Chimonanthus campanulatus* R.H. Chang & C.S. Din [30], *Juglan regia* L. and *Juglans sigillata* Dode [31], and *Hibiscus mutabilis* L. [32]. The probe of the 5S rDNA fragment 5′-TCAGAACTCCGAAGTTAAGCGTGCTTGGGCGAGGTAGTAC-3′ was designed and developed in *Piptanthus concolor* Harrow ex Craib [33], *Zanthoxylum armatum* Candelle [34], *B. diaphana* and *B. soulieana* [28], *F. pennsylvanica*, *S. oblata*, *L. lucidum*, *L.* × *vicaryi* [29], *Ch. campanulatus* [30], *J. regia* and *J. sigillata* [31], and *H. mutabilis* [32]. The probe of the (TTG)_6_ trinucleotide repeat sequence 5′-TTGTTGTTGTTGTTGTTG-3′ was designed and developed in *Avena* L. species [35], *F. pennsylvanica*, *S. oblata*, *L. lucidum*, and *L.* × *vicaryi* [29]. All three oligo-probes were synthesized by Sangon Biotech Co., Ltd. (Shanghai, China) and first tested in *H. rhamnoides* simultaneously in a single round of FISH. The oligo-probes were 5′-labeled with 6-carboxyfluorescein or 6-carboxytetramethylrhodamine.

### 2.2. FISH and Karyotype Analysis

Root tips were cut from *H. rhamnoides* seedlings and treated with nitrous oxide gas for 3 h, fixed in acetic acid for approximately 10 min, and finally preserved in 75% ethanol for further chromosome preparation. The root tip slides were prepared according to the method described by Luo et al. [33]. The meristematic zone (~1 mm) of the root tip was digested with pectinase and cellulase (Yakult Pharmaceutical Industry Co., Ltd., Tokyo, Japan) and then suspended; this suspension was used for slide preparation using the drop method. Chromosomes were denatured for 2 min at 80 °C and hybridized with oligo-probes for 2 h at 37 °C using the method described by Luo et al. [33]. After counterstaining with 4,6-diamidino-2-phenylindole (DAPI) containing VECTASHIELD Antifade Mounting Medium (Vector Laboratories, Inc., Burlingame, CA, USA) and covering with a coverslip, the slides were observed under an Olympus BX-63 microscope (Olympus Corporation, Tokyo, Japan). FISH photomicrographs were obtained using a DP-70 CCD camera connected to the BX-63 microscope. Chromosome spreads in raw images were processed with DP Manager (Olympus Corporation, Tokyo, Japan) and Photoshop CC 2015 (Adobe Systems Incorporated, San Jose, CA, USA). Approximately 90 mitotic metaphases or mitotic metaphase to anaphases from 30 slides of 15 *H. rhamnoides* root tips were observed. More than 10 cells in the mitotic metaphase or mitotic metaphase to anaphase with good chromosome spread were used to count the chromosomes. Three high-quality spreads were used for karyotype analysis. All chromosomes were aligned by length, from the longest to shortest. The chromosome ratio was determined as the length of the longest chromosome to that of the shortest chromosome.

## 3. Results

### 3.1. FISH-Enabled Visualization of H. rhamnoides Chromosomes

**Figure 1:**
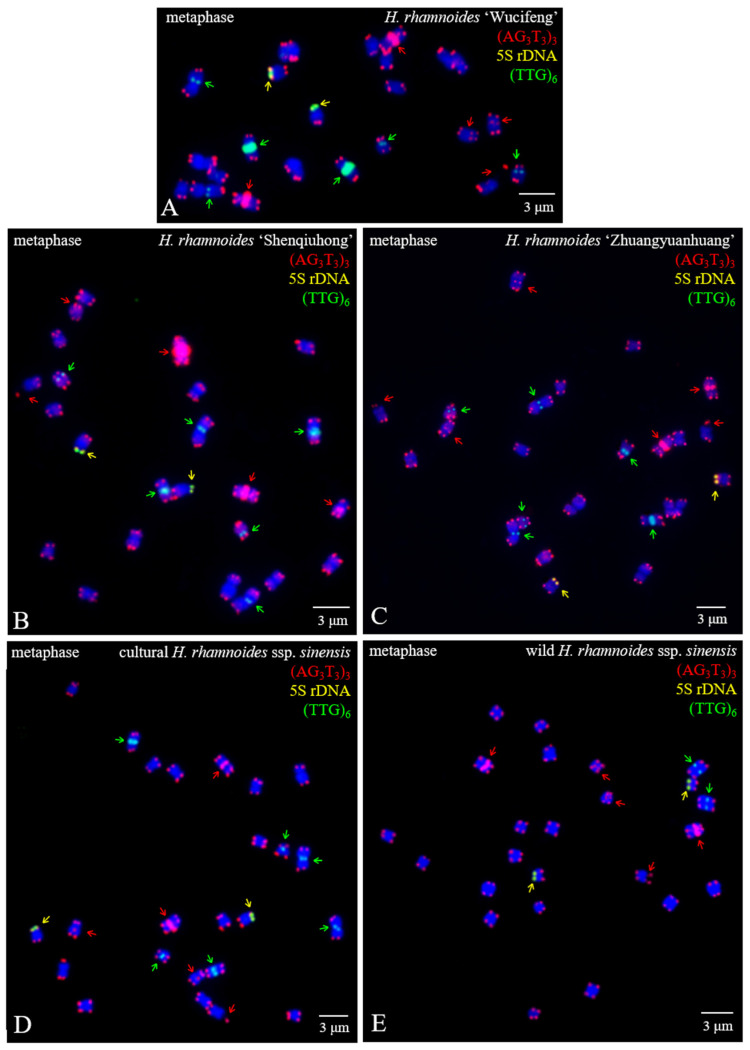
Mitotic metaphase chromosomes of *Hippophaë rhamnoides* detected using (AG_3_T_3_)_3_ (red), 5S rDNA (yellow), and (TTG)_6_ (green). (**A**) *Hippophaë rhamnoides* ‘Wucifeng’, (**B**) *H. rhamnoides* ‘Shenqiuhong’, (**C**) *H. rhamnoides* ‘Zhuangyuanhuang’, (**D**) cultural *H. rhamnoides* ssp. *sinensis*, and (**E**) wild *H. rhamnoides* ssp. *sinensis*. Red arrows show (AG_3_T_3_)_3_ located at the interstitial region of a chromosome or at the telomere region far away from the chromosome end, whereas yellow arrows show 5S rDNA and green show (TTG)_6_. (AG_3_T_3_)_3_ located at the chromosome end is not indicated with an arrow. The blue chromosomes were counterstained by DAPI. Scale bar = 3 μm.

**Figure 2:**
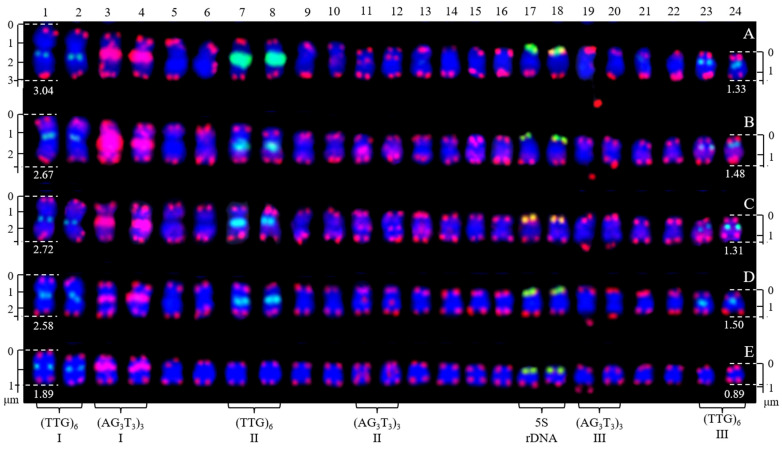
Chromosomes from Figure 1 presented individually. The chromosomes were aligned by a combination of length from the longest to shortest and signal pattern. The left/right number represents the chromosome length: (**A**) *H. rhamnoides* ‘Wucifeng’, 3.04–1.33 μm; (**B**) *H. rhamnoides* ‘Shenqiuhong’, 2.67–1.48 μm; (**C**) *H. rhamnoides* ‘Zhuangyuanhuang’, 2.72–1.31 μm; (**D**) cultural *H. rhamnoides* ssp. *sinensis*, 2.58–1.50 μm; and (**E**) wild *H. rhamnoides* ssp. *sinensis*, 1.89–0.89 μm. The numbers on the top represent the chromosome numbers, whereas the bottom probes labeled some chromosomes: chromosomes 1/2, 7/8, 23/24 were labeled by (TTG)_6_ I (**A**–**E**), (TTG)_6_ II (**A**–**D**), and (TTG)_6_ III (**A**–**D**), whereas chromosomes 3/4, 11/12, and 19/20 were labeled by (AG_3_T_3_)_3_ I (**A**–**E**), (AG_3_T_3_)_3_ II (**A**–**E**), and (AG_3_T_3_)_3_ III (**A**–**E**); chromosomes 17/18 (**A**–**E**) were labeled by 5S rDNA.

The mitotic metaphase of five *H. rhamnoides* taxa detected using (AG_3_T_3_)_3_, 5S rDNA, and (TTG)_6_ is illustrated in Figure 1. To visualize FISH signal distribution, each chromosome was cut from Figure 1 and aligned in Figure 2 based on its length and signal pattern. A total of 24 chromosomes were observed in each taxon of *H. rhamnoides* ‘Wucifeng’ (Figure 1A and Figure 2A), *H. rhamnoides* ‘Shenqiuhong’ (Figure 1B and Figure 2B), *H. rhamnoides* ‘Zhuangyuanhuang’ (Figure 1C and Figure 2C), cultural *H. rhamnoides* ssp. *sinensis* (Figure 1D and Figure 2D), and wild *H. rhamnoides* ssp. *sinensis* (Figure 1E and Figure 2E). The chromosome size of each *H. rhamnoides* taxon was 1.33–3.04 μm for *H. rhamnoides* ‘Wucifeng’, 1.48–2.67 μm for *H. rhamnoides* ‘Shenqiuhong’, 1.31–2.72 μm for *H. rhamnoides* ‘Zhuangyuanhuang’, 1.50–2.58 μm for cultural *H. rhamnoides* ssp. *sinensis*, and 0.89–1.89 μm for wild *H. rhamnoides* ssp. *sinensis*. The size ranged from 0.89 to 3.03 μm, which is similar to that of small chromosomes. The ratio of the longest to shortest chromosomes in the mitotic metaphase was 3.40, indicating karyotype asymmetry in *H. rhamnoides*. Owing to the unclear centromeres of most chromosomes and their small size, the short and long arms of the chromosomes were not well characterized for further karyotype analysis.

(AG_3_T_3_)_3_ was located not only at the end of each chromosome but also at four chromosomally proximal regions (chromosomes 3/4/11/12); it was even dissociated from one end of chromosome 19 (satellite bodies) in five *H. rhamnoides* taxa (Figure 1). Two strong signals of (AG_3_T_3_)_3_ were observed in the proximal region of chromosome 3/4, whereas the other chromosomes showed minor differences in (AG_3_T_3_)_3_ signal intensity in five *H. rhamnoides* taxa (Figure 2). (TTG)_6_ was observed at six chromosomally proximal regions (chromosome 1/2/7/8/23/24) in three cultivars *H. rhamnoides* ‘Wucifeng’ (Figure 1A and Figure 2A), *H. rhamnoides* ‘Shenqiuhong’ (Figure 1B and Figure 2B), and *H. rhamnoides* ‘Zhuangyuanhuang’ (Figure 1C and Figure 2C) and one cultural *H. rhamnoides* ssp. *sinensis* (Figure 1D and Figure 2D), but only at two chromosomally proximal regions (chromosome 1/2) in wild *H. rhamnoides* ssp. *sinensis* (Figure 1E and Figure 2E). Two strong signals of (TTG)_6_ were observed in the chromosomally proximal region of two chromosomes (chromosome 7/8) in three cultivars of *H. rhamnoides* (Figure 1A–C and Figure 2A–C) and one cultural *H. rhamnoides* ssp. *sinensis* (Figure 1D and Figure 2D), whereas the other chromosomes showed minor differences in (TTG)_6_ signal intensity in five *H. rhamnoides* taxa (Figure 1A–E and Figure 2A–E). The 5S rDNA nearly overlapped with (AG_3_T_3_)_3_ in two chromosome ends (chromosome 17/18) in five *H. rhamnoides* taxa (Figure 1A–E and Figure 2A–E), and the signal intensity showed minor differences.

**Figure 3:**
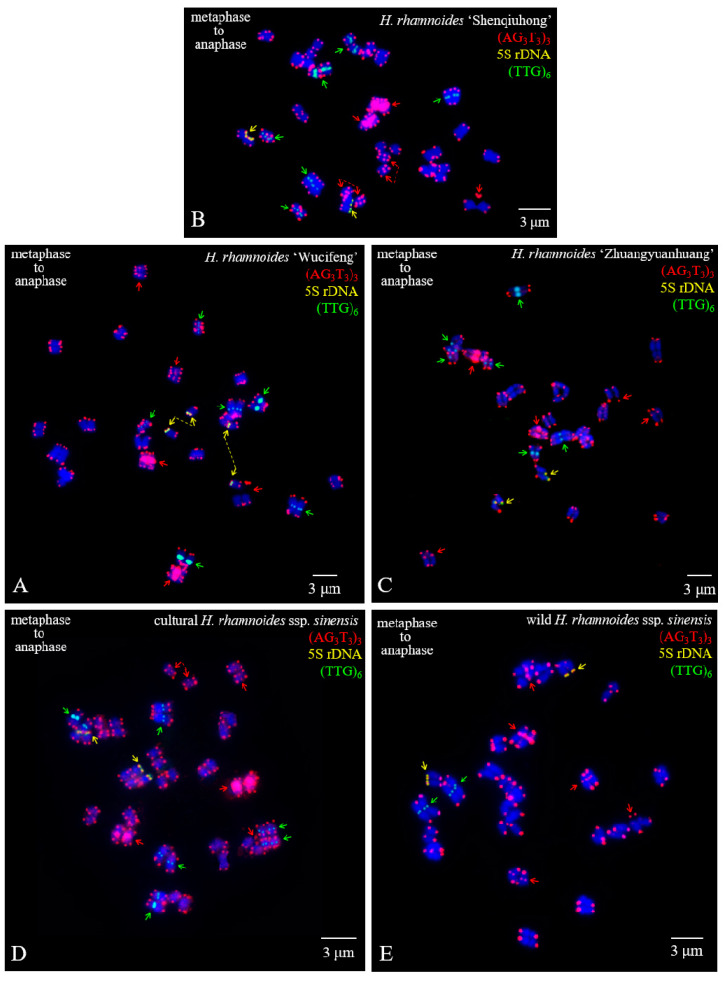
Mitotic metaphase to anaphase chromosomes of *Hippophaë rhamnoides* detected using (AG_3_T_3_)_3_ (red), 5S rDNA (yellow), and (TTG)_6_ (green). (**A**) *Hippophaë* *rhamnoides* ‘Wucifeng’, (**B**) *H. rhamnoides* ‘Shenqiuhong’, (**C**) *H. rhamnoides* ‘Zhuangyuanhuang’, (**D**) cultural *H. rhamnoides* ssp. *sinensis,* and (**E**) wild *H. rhamnoides* ssp. *sinensis*. Red arrows show (AG_3_T_3_)_3_ located at the interstitial region of chromosomes or telomere region far away from the chromosome end, whereas yellow arrows show 5S rDNA and green arrows show (TTG)_6_. (AG_3_T_3_)_3_ located at the chromosome end has not been indicated with an arrow. Dotted lines connecting arrows represent two chromosomes split from one chromosome. We did not annotate all split chromosomes; we only annotated 4 chromosomes in Figure 3A (yellow dotted line), 4 chromosomes in Figure 3B (red dotted line), and 2 chromosomes in Figure 3D (red dotted line). The blue chromosomes were counterstained by DAPI. Scale bar = 3 μm.

**Figure 4:**
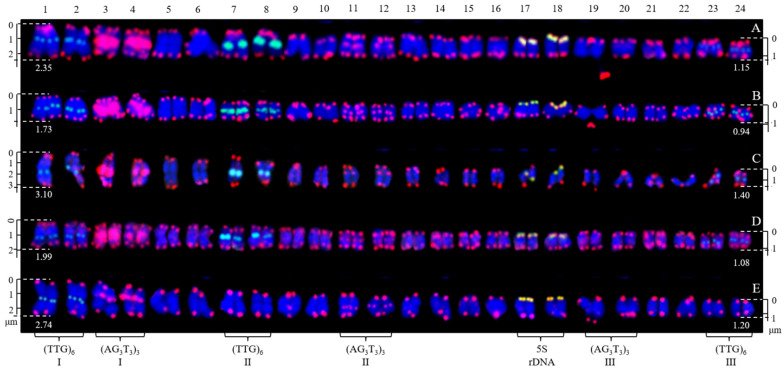
Chromosomes from Figure 2 presented individually. The chromosomes were aligned by a combination of length from the longest to shortest and signal pattern. The left/right number represents the chromosome length: (**A**) *H. rhamnoides* ‘Wucifeng’, 2.35–1.15 μm; (**B**) *H. rhamnoides* ‘Shenqiuhong’, 1.73–0.94 μm; (**C**) *H. rhamnoides* ‘Zhuangyuanhuang’, 3.10–1.40 μm; (**D**) cultural *H. rhamnoides* ssp. *sinensis*, 1.99–1.08 μm; and (**E**) wild *H. rhamnoides* ssp. *sinensis*, 2.74–1.20 μm. The numbers on the top represent the chromosome number, whereas the bottom probes labeled some chromosomes: chromosomes 1/2, 7/8, 23/24 were labeled by (TTG)_6_ I (**A**–**E**), (TTG)_6_ II (**A**–**D**), and (TTG)_6_ III (**A**–**D**); chromosomes 3/4, 11/12, 19/20 were labeled by (AG_3_T_3_)_3_ I (**A**–**E**), (AG_3_T_3_)_3_ II (**A**–**E**), and (AG_3_T_3_)_3_ III (**A**–**E**); and chromosomes 17/18 (**A**–**E**) were labeled by 5S rDNA.

The mitotic metaphase to anaphase chromosomes of five *H. rhamnoides* taxa detected using (AG_3_T_3_)_3_, 5S rDNA, and (TTG)_6_ are illustrated in Figure 3. To clearly display FISH signal distribution, each chromosome was cut from Figure 3 and aligned in Figure 4 based on its length, signal pattern, and chromosome segregation. The chromosome size of each *H. rhamnoides* taxon was 1.15–2.35 μm for *H. rhamnoides* ‘Wucifeng’ (Figure 3A and Figure 4A), 0.94–1.73 μm for *H. rhamnoides* ‘Shenqiuhong’ (Figure 3B and Figure 4B), 1.40–3.10 μm for *H. rhamnoides* ‘Zhuangyuanhuang’ (Figure 3C and Figure 4C), 1.08–1.99 μm for cultural *H. rhamnoides* ssp. *sinensis* (Figure 3D and Figure 4D), and 1.20–2.74 μm for wild *H. rhamnoides* ssp. *sinensis* (Figure 3E and Figure 4E). The size ranged from 0.94 to 3.10 μm, which is similar to that of small chromosomes. The ratio of the longest to shortest chromosomes in the mitotic metaphase to anaphase was 3.30, indicating karyotype asymmetry in *H. rhamnoides*. Due to chromosome segregation in the mitotic metaphase to anaphase, chromosome numbers in each taxon in Figure 3 ranged from 24 to 48. Several of them have been split into two separate chromosomes and far away at a certain distance (to make them easy to count, e.g., in Figure 3A,B,D, shown by the dotted line), whereas most of them were closely matched to each other (which makes it difficult to determine whether there is one or two chromosomes) in Figure 3. The signal number and intensity of (AG_3_T_3_)_3_, 5S rDNA, and (TTG)_6_ mitotic metaphase to anaphase chromosomes were nearly consistent with those of mitotic metaphase chromosomes if the two split chromosomes were integrated as one unit (Figure 4). Owing to the cryptic centromeres of several chromosomes and their small size, the short and long arms of the chromosomes were not well characterized for further karyotype analysis.

### 3.2. Physical Map Distinguished Chromosomes

**Figure 5:**
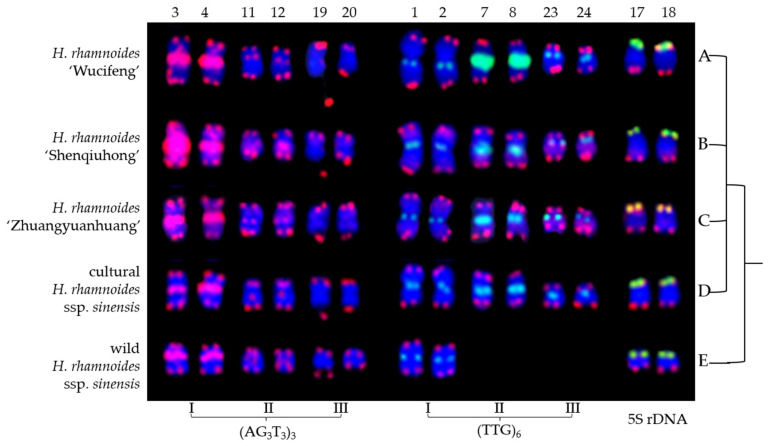
Chromosomes of *Hippophaë rhamnoides* identified using (AG_3_T_3_)_3_, (TTG)_6_, and 5S rDNA cut from Figure 3. (A) *Hippophaë rhamnoides* ‘Wucifeng’, (**B**) *H. rhamnoides* ‘Shenqiuhong’, (**C**) *H. rhamnoides* ‘Zhuangyuanhuang’, (**D**) cultural *H. rhamnoides* ssp. *sinensis*, and (**E**) wild *H. rhamnoides* ssp. *sinensis*. The numbers on the upper side represent the chromosome number consistent with *H. rhamnoides* in Figure 3. All chromosomes exhibited (AG_3_T_3_)_3_ end signals (red), whereas chromosome 3/4, 11/12 exhibited interstitial telomere repeat (AG_3_T_3_)_3_ I, (AG_3_T_3_)_3_ II signals in (**A**–**E**) (red), and chromosome 19 exhibited (AG_3_T_3_)_3_ III end signals far away from the chromosome ends in (**A**–**E**) (red). Chromosome 1/2 exhibited (TTG)_6_ I signal in (**A**–**E**) (green), whereas chromosome 7/8, 23/24 exhibited (TTG)_6_ II, (TTG)_6_ III signals in (**A**–**D**) (green). Chromosome 17/18 exhibited 5S rDNA signals in (**A**–**E**) (yellow). Figure 5 only exhibits chromosomes with (AG_3_T_3_)_3_, (TTG)_6_, and 5S rDNA signals, exclusively identified chromosomes, whereas Figure 5 does not present chromosomes with no diagnostic chromosome signals, such as chromosomes only with (AG_3_T_3_)_3_ end signal. Therefore, Figure 5 is a simplified version of Figure 3.

**Figure 6:**
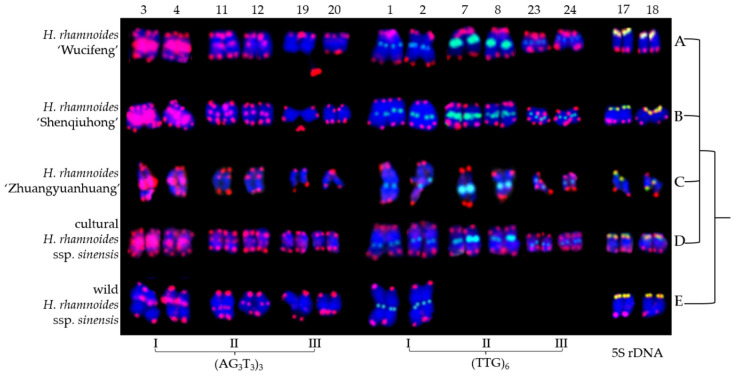
Chromosomes of *Hippophaë rhamnoides* identified using (AG_3_T_3_)_3_, (TTG)_6_, and 5S rDNA cut from Figure 4. (**A**) *Hippophaë rhamnoides* ‘Wucifeng’, (**B**) *H. rhamnoides* ‘Shenqiuhong’, (**C**) *H. rhamnoides* ‘Zhuangyuanhuang’, (**D**) cultural *H. rhamnoides* ssp. *sinensis*, and (**E**) wild *H. rhamnoides* ssp. *sinensis*. The numbers on the upper side represent chromosome number consistent with *H. rhamnoides* in Figure 4. All chromosomes exhibited (AG_3_T_3_)_3_ end signals (red), whereas chromosome 3/4 and 11/12 exhibited interstitial telomere repeat (AG_3_T_3_)_3_ I, (AG_3_T_3_)_3_ II signals in (**A**–**E**) (red), and chromosome 19 exhibited (AG_3_T_3_)_3_ III end signals far away from the chromosome ends in (**A**–**E**) (red). Chromosome 1/2 exhibited (TTG)_6_ I signal in (**A**–**E**) (green), whereas chromosome 7/8, 23/24 exhibited (TTG)_6_ II, (TTG)_6_ III signals in (**A**–**D**) (green). Chromosome 17/18 exhibited 5S rDNA signals in (yellow). Figure 5 only exhibits chromosomes with (AG_3_T_3_)_3_, (TTG)_6_, and 5S rDNA signals, exclusively identified chromosomes, whereas Figure 6 does not present chromosomes with no diagnostic chromosome signals, such as chromosomes only with (AG_3_T_3_)_3_ end signal. Therefore, Figure 6 is a simplified version of Figure 4.

**Figure 7:**
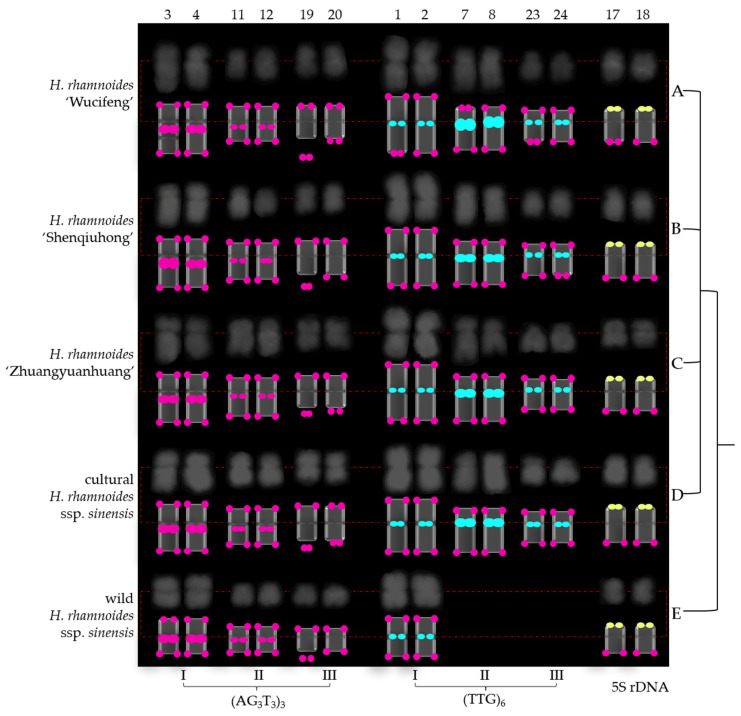
Physical map of *Hippophaë rhamnoides*. (**A**) *Hippophaë rhamnoides* ‘Wucifeng’, (**B**) *H. rhamnoides* ‘Shenqiuhong’, (**C**) *H. rhamnoides* ‘Zhuangyuanhuang’, (**D**) cultural *H. rhamnoides* ssp. *sinensis*, and (**E**) wild *H. rhamnoides* ssp. *sinensis*. In order to better exhibit the centromere location, each chromosome in black–white was another version of the chromosome in blue in Figure 5. The red dotted line indicates centromere location. Small chromosomes with dim centromere location were aligned by the subtle clues and traces of chromosome white/black contrast. Therefore, determination of their centromere location is difficult. The signal pattern ideograms were constructed based on the above black–white chromosome and signal patterns of chromosomes in Figure 5. The numbers on the upper side represent chromosome number, and the (AG_3_T_3_)_3_, (TTG)_6_, and 5S rDNA signal types at the bottom are consistent with *H. rhamnoides* in Figure 5.

**Figure 8:**
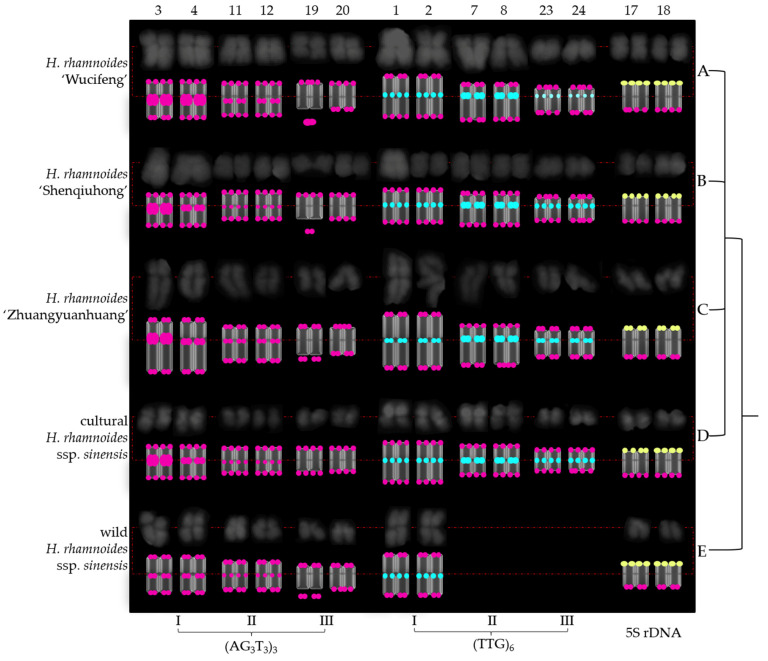
Physical map of *Hippophaë rhamnoides*. (**A**) *Hippophaë rhamnoides* ‘Wucifeng’, (**B**) *H. rhamnoides* ‘Shenqiuhong’, (**C**) *H. rhamnoides* ‘Zhuangyuanhuang’, (**D**) cultural *H. rhamnoides* ssp. *sinensis*, and (**E**) wild *H. rhamnoides* ssp. *sinensis*. In order to better exhibit the centromere location, each chromosome in black–white was another version of the chromosome in blue in Figure 6. The red dotted line indicates centromere location. Small chromosomes with dim centromere location were aligned by the subtle clues and traces of chromosome white/black contrast. Therefore, determination of their centromere location is difficult. The signal pattern ideograms were constructed based on the above black–white chromosome and signal patterns of chromosomes in Figure 6. The numbers on the upper side represent chromosome number and the (AG_3_T_3_)_3_, (TTG)_6_, and 5S rDNA signal types at the bottom are consistent with *H. rhamnoides* in Figure 6.

Next, as shown in Figure 5 and Figure 6, the chromosomes were further eliminated with a common signal. As a result, the chromosomes of *H. rhamnoides* identified by (AG_3_T_3_)_3_, (TTG)_6_, and 5S rDNA were aligned into a simplified version of Figure 3 and Figure 4. To better exhibit the centromere location, each chromosome was visualized in a black–white version (Figure 7 and Figure 8). The signal pattern ideograms were constructed based on the above black–white visualization of the chromosomes and their signal patterns in Figure 5 and Figure 6. A clear centromere location was observed in chromosomes 1/2, 3/4 in all five *H. rhamnoides* taxa. Generally, chromosome 3 of *H. rhamnoides* ‘Wucifeng’ was seen as a dicentric chromosome (Figure 7). The chromosome 1/2, 3/4 arm ratio ranged from 1 to 1.7; hence, the two chromosomes have been designated as median region (m, 1 < r < 1.7). The symmetry of chromosome 1/2 was higher than that of chromosome 3/4. The centromere location was also observed for a few other chromosomes, such as chromosome 7/8, 19/20 of *H. rhamnoides* ’Zhuangyuanhuang’, albeit not as clearly as that of chromosomes 1/2, 3/4. It was difficult to determine the centromere location of other chromosomes as they were small in size and had lightly stained centromeres, which also made it difficult to count their arm ratios and construct a karyotype formula.

Owing to the lack of effective discernment, (AG_3_T_3_)_3_ located at the end of each chromosome was ignored here. Three (AG_3_T_3_)_3_ signal types identified six chromosomes of *H. rhamnoides* (Figure 5, Figure 6, Figure 7 and Figure 8). Type I (AG_3_T_3_)_3_ discerned chromosome 3/4 by two strong signals in the proximal region. Type II (AG_3_T_3_)_3_ discerned chromosome 11/12 by two small signals in the proximal region. Type III (AG_3_T_3_)_3_ discerned chromosome 19 by a signal-dissociated chromosome end (satellite body). Chromosome 20 could not be discerned well based on its match with chromosome 19 (chromosome length, arm, centromere, and common signal).

(TTG)_6_ also showed three types of signal patterns (Figure 5, Figure 6, Figure 7 and Figure 8). Type I (TTG)_6_ discerned chromosome 1/2 by two small signals in the proximal region in five *H. rhamnoides* taxa (Figure 5, Figure 6, Figure 7 and Figure 8). Type II (TTG)_6_ discerned chromosome 7/8 by two strong signals in the proximal region in three cultivars *H. rhamnoides* ‘Wucifeng’ (Figure 5A, Figure 6A, Figure 7A and Figure 8A), *H. rhamnoides* ‘Shenqiuhong’ (Figure 5B, Figure 6B, Figure 7B and Figure 8B), and *H. rhamnoides* ‘Zhuangyuanhuang’ (Figure 5C, Figure 6C, Figure 7C and Figure 8C), and cultural *H. rhamnoides* ssp. *sinensis* (Figure 5D, Figure 6D, Figure 7D and Figure 8D). Type III (TTG)_6_ discerned chromosome 17/18 by two small signals in the proximal region (Figure 5, Figure 6, Figure 7 and Figure 8). Consequently, (TTG)_6_ may distinguish wildtype *H. rhamnoides* ssp. *sinensis* from three cultivars: *H. rhamnoides* ‘Wucifeng’, *H. rhamnoides* ‘Shenqiuhong’, and *H. rhamnoides* ‘Zhuangyuanhuang’, and cultural *H. rhamnoides* ssp. *sinensis*. Therefore, (AG_3_T_3_)_3_ and (TTG)_6_ are diverse and effective for chromosome recognition and taxon identification in *H. rhamnoides*. 5S rDNA discerned chromosome 17/18 by two small overlapping signals of (AG_3_T_3_)_3_ and 5S rDNA in one chromosome end (Figure 5, Figure 6, Figure 7 and Figure 8). 5S rDNA only discerned two chromosomes that were conserved in five *H. rhamnoides* taxa.

Overall, (AG_3_T_3_)_3_, (TTG)_6_, and 5S rDNA may discern 14 chromosomes in five *H. rhamnoides* taxa. More importantly, the combination of the three oligo-probes may identify one wild *H. rhamnoides* taxon from four *H. rhamnoides* cultivars.

## 4. Discussion

### 4.1. Karyotype Analysis

Chromosome number and morphological characteristics are important components of karyotypes. *H. rhamnoides* chromosomes in the mitotic metaphase are small (3.04–0.89 μm), and most of them showed a similar morphology in the current study. Owing to the small size of the chromosome and equivocal centromere of half chromosomes, we only measured total chromosome size here. The length of the long/short arm, karyotype, and cytotype, which are conventionally assessed in karyotype analysis, could not be determined in this study. Studies have reported the chromosome size of four *Hippophaë* taxa: 1.67–4.44 μm [36], 2.6–5.2 μm [37], and 0.97–2.77 μm [38] in *H. rhamnoides* ssp. *sinensis*; 1.00–2.85 μm in *H. rhamnoides* L. ssp. *turkestanica* Rousi [38]; 0.77–2.84 μm in *H. thibetana* Schlechtend [38]; and 0.57–2.81 μm in *H. neurocarpa* S.W. Liu et T.N [38]. The chromosome size that we reported (0.89–3.03 μm) is within the range specified by previous studies on *Hippophaë* taxa (0.77–5.2 μm). Several chromosome sizes of other woody plants have been published: 1.05–1.81 μm in *L. lucidum*, 1.12–2.06 μm in *F. pennsylvanica*, 1.50–2.32 μm in *S. oblata* [29], 0.97–2.16 μm in *J. regia* [31], 1.23–2.34 μm in *Z. armatum* [34], 1.07–2.41 μm in *Ch. campanulatus* [30], 1.82–2.85 μm in *B. diaphana* [28], 1.18–3.0 μm in *H. mutabilis* [32], 1–4 μm in *Citrus* species [39], and 4.03–7.21 μm in *P. concolor* [33]. The chromosome size in our study (0.89–3.03 μm) is close to that of *H. mutabilis* (1.18–3.0 μm). Chromosome size is controlled by the chromosome phase when slide preparation is disturbed by measurements. As a result, chromosome size may be a guide for not only qualitative analysis (such as small chromosomes), but also quantitative analysis in chromosome research.

As observed in the present study, 24 chromosomes were counted in five *H. rhamnoides* taxa, which is in accordance with the known number (2n = 24) represented in older cytogenetic analyses [17,36,37,38,40,41,42,43], but different from the results (2n = 12) of Borodina [44] and Darmer [45]. This result (x = 12) is also in accordance with the known basic number ranging from 11 to 14 [46].

Satellite bodies, as hereditary features, may be used to identify chromosomes and distinguish species [47,48]. One pair of *H. rhamnoides* taxon satellite chromosomes was observed in previous studies [36,37,38], whereas Liang et al. [40] observed three pairs of *H. rhamnoides* subsp. *sinensis* satellite chromosomes. However, Li et al. [41] did not observe satellite chromosomes in *H. rhamnoides* taxon. Interestingly, only one satellite body was clearly observed in the present study. The possible reasons are as follows: (1) the other satellite body was too close to the chromosome arm to be well discovered; (2) the other satellite body was lost during slide preparation; (3) the *H. rhamnoides* chromosome was small in size, causing the satellite body to be smaller; (4) the satellite body is a fickle structure; hence, translocation and transfer of the satellite body occurs readily; and (5) the inconsistent evolution of two satellite bodies caused the other one to lack the portion that is visualized by oligo-probes. These possibilities may cause a change in the number of satellite bodies.

### 4.2. Role of (AG_3_T_3_)_3_, (TTG)_6_, and 5S rDNA

(AG_3_T_3_)_3_, a classic chromosome end marker, is typically located in the distal region of the chromosome in *H. mutabilis* [32], *J. regia*, *J. sigillata* [31], *F. pennsylvanica*, *S. oblata*, *L. lucidum*, *L.* × *vicaryi* [28], *B. diaphana*, and *B. soulieana* [28]. Other similar types of (T_x_A_y_G_z_)_n_ [49] have also been identified at each chromosome end in the woody plants *C. sinensis* × *P. trifoliata* [23], *Citrus clementina* Hort. Ex Tan. [50], *Dendropanax morbiferus* H. Lév., *Eleutherococcus sessiliflorus* (Rupr. Et Maxim.) Seem., *Kalopanax septemlobus* (Thunb. ex A.Murr.) Koidz [51], *Ginkgo biloba* L., *Hordeum vulgare* L., *Phaseolus vulgaris* sensu Blanco, non L. and *Trigonella foenum*-*graecum* L. [52], *Rosa wichurana* Cr‚p. [53], *Cestrum elegans* (Brongn. ex Neumann) Schltdl. [54], *Pinus* L. species [26], and *Podocarpus* L’Hér. ex Pers. species [55]. The (AG_3_T_3_)_3_ distal signal is generally ineffective in distinguishing chromosomes; however, it ensures chromosome integrity via a two-end signal, thereby guaranteeing accurate counts of chromosome number in previous studies. Similarly, in this study, (AG_3_T_3_)_3_ detected all chromosomes by FISH signal location at the chromosome termini and ensured the accuracy of chromosome counts of *H. rhamnoides*.

Occasionally, (AG_3_T_3_)_3_ or other similar types deviated from the end and were observed in the proximal and interstitial regions of chromosomes in the woody plant *Ci. sinensis* × *P. trifoliata* [23], *Ch. campanulatus* [30], *Aralia elata* (Miq.) Seem. [51], *Pinus densiflora* Siebold & Zucc. [52], *R. wichurana* [53], *Cestrum parqui* Benth. and *Vestia foetida* (Ruiz & Pav.) Hoffmanns. [56,57], and *Podocarpus* L. Her. ex Persoon species [55]. Furthermore, (AG_3_T_3_)_3_ dissociated from the chromosome (location satellite bodies) and was observed in *Ch. campanulatus* [30]. The distal, proximal, and dissociated signals of (AG_3_T_3_)_3_ have confirmed that it was easily distinguished in previous studies. Similarly, in this study, (AG_3_T_3_)_3_ detected six chromosomes by different FISH signal locations at the distal, proximal, and dissociated (location satellite bodies) chromosomes of *H. rhamnoides*.

(TTG)_6_, as a useful non-chromosome end marker, has demonstrated abundant variation in 16 *Avena* species [35], *F. pennsylvanica*, *S. oblata*, *L. lucidum*, and *L.* × *vicaryi* [29]. The signal location moved from the subterminal region to the proximal region, whereas the signal intensity ranged from weak and small to strong and large. The signal band on one chromosome ranged from one to more. Research on (TTG)_n_ as an oligo-FISH marker is scarce. However, (TTG)_10_ has also emerged as an important microsatellite for genetic marker characterization in *Capsicum annuum* L. [58], *Triticum aestivum* L. [59], and *Nicotiana tabacum* L. [60]. In the present study, (TTG)_6_ sites in *H. rhamnoides* were relatively stable and were only located in the proximal region; nevertheless, the signal strength changed from weak to strong, similar to that in *Avena* species and Oleaceae species. Moreover, our results revealed variability in the number of (TTG)_6_ among *H. rhamnoides* taxa that showed divergence (two sites in wild *H. rhamnoides* ssp. *sinensis*, but six sites in the other four *H. rhamnoides* cultivars), which also agreed with the varied (TTG)_6_ distribution among *Avena* species and Oleaceae species. Therefore, (TTG)_6_ is an effective oligo-FISH marker for detecting species or subspecies.

5S rDNA has been used extensively as a chromosome marker and exhibits substantial conservation and stability in woody plants *Annona cherimola* L. [61], *C. sinensis* × *P. trifoliata* [23], *A. elata*, *D. morbiferus*, *E. sessiliflorus*, *K. septemlobus* [51], *Ch. campanulatus* [30], *G. biloba* and *P. densiflora* [52], *H. rhamnoides* [17], *R. wichurana* [53], *Passiflora* species [62], *Cestrum* species [56], and *V. foetida* [57]. However, 5S rDNA has also showed high diversity in other plants, including *A. hypogaea* [20], *Fragaria* L. species [63], *Crocus sativus* L., *Crocus vernus* (L.) Hill [64,65], and *P. concolor* [33].

In the current study, 5S rDNA nearly colocalized with (AG_3_T_3_)_3_ at the two chromosome ends. Similar colocalization has been found in *B. diaphana* [28] and *Chrysanthemum zawadskii* (Herb.) Tzvel. [66]. Puterova et al. [17] also found two 5S rDNA terminal signals in *H. rhamnoides* chromosome, which supports the results of the present study. The 5S rDNA distribution in the termini has also been reported in *F. pennsylvanica*, *S. oblata*, *L. lucidum*, *L.* × *vicaryi* [29], and *P. foetida* [62]. The FISH results presented herein confirm a substantial conservation in the number and location of 5S rDNA among *H. rhamnoides* taxa. As a consequence, the present study results indicate that 5S rDNA cannot clearly distinguish *H. rhamnoides* taxa.

### 4.3. Detection of the X/Y-Chromosome in H. rhamnoides

The large X and small Y chromosomes in *H. rhamnoides* were revealed by Shchapov [43]. Another cytogenetic study on *H. rhamnoides* female karyotype without determination of sex chromosomes was conducted by Rousi and Arohonka [42]. However, Puterova et al. [17] successfully identified the X/Y-chromosome in *H. rhamnoides* using FISH from repetitive genomic DNA sequences. Unfortunately, we were unable to differentiate sex chromosomes and autosomes in the present study. Nevertheless, according to the previous analysis of chromosome spreads [17,38,43], the X-chromosome is one of the three longest pairs (chromosome 1–6), and the Y-chromosome is one of the five shortest pairs (chromosome 15–24). Considering the similar lengths of chromosomes 5/6 and 7/8 in the present study, the X-chromosome is one of the four longest pairs (chromosome 1–8) here. In addition, 5S rDNA is located in the autosome [17]. In the current FISH mapping, chromosomes 1/2, 7/8, and 23/24 showed (TTG)_6_ I, (TTG)_6_ II, and (TTG)_6_ III signals; chromosome 3/4 and 19/20 showed (AG_3_T_3_)_3_ I and (AG_3_T_3_)_3_ III signals; and chromosome 17/18 showed 5S rDNA signals. In other words, the X-chromosome was labeled by (TTG)_6_ I, (TTG)_6_ II, or (AG_3_T_3_)_3_ I, whereas the Y-chromosome was labeled by (TTG)_6_ III or (AG_3_T_3_)_3_ III. Previous work has also identified sex chromosomes using 5S rDNA and telomeric (CCCTAA)_3_ in *Humulus japonicus* Siebold & Zucc. [67], 5S rDNA, 45S rDNA, and the sex chromosome repetitive DNA sequences in *Spinacia oleracea* L. [68].

## 5. Conclusions

To the best of our knowledge, this is the first study to assess (AG_3_T_3_)_3_, (TTG)_6_, and 5S rDNA in *H. rhamnoides*. This study was conducted to identify the chromosomes of *H. rhamnoides* and compare cultural/wild *H. rhamnoides* ssp. *sinensis* with three varieties of *H. rhamnoides*. Information on chromosome identification, as well as the identification of taxa, will not only help elucidate visual and elaborate physical mapping but will also guide breeders’ utilization of wild resources of *H. rhamnoides*. The use of the oligo-FISH system will enable, for the first time in the genomics era, a comprehensive cytogenetic analysis in *H. rhamnoides*. The results of this study will help identify chromosomes and establish physical maps of other *Hippophaë* taxa and close genera. We are committed to developing additional oligos (such as detection centromeres) to generate a high-resolution and informative cytogenetic map of the genome regions of *H. rhamnoides*.

## Data Availability

All data and materials are included in the form of graphs in this article.
